# Physical activity and screen time in out of school hours care: an observational study

**DOI:** 10.1186/s12887-019-1653-x

**Published:** 2019-08-14

**Authors:** Carol Maher, Rosa Virgara, Tony Okely, Rebecca Stanley, Millie Watson, Lucy Lewis

**Affiliations:** 1 School of Health Sciences, University of South Australia, Adelaide, SA 5000 Australia; 2 Faculty of Social Sciences, University of Wollongong, Wollongong, NSW 2522 Australia; 3 College of Nursing and Health Sciences, Flinders University, Bedford Park, SA 5042 Australia

**Keywords:** Exercise, Television, Videogames, Sedentary behaviour, Child, Children, Childcare, After school care

## Abstract

**Background:**

This study aimed to describe, and identify predictors of, physical activity and screen time in children attending out of school hours care (OSHC).

**Method:**

Twenty-three randomly selected OSHC centres (*n* = 1068 children) participated in this observational, cross-sectional study. Service directors completed interviews regarding policy, training, scheduling and equipment related to physical activity and screen time. Children’s activity behaviours (moderate to vigorous physical activity (MVPA), light physical activity, sedentary time and screen time) were measured using standardised direct observation.

**Results:**

Directors’ interviews revealed a lack of formal policy guiding physical activity and screen time. Time spent in activity behaviours varied widely among OSHC services; for example, average time spent in MVPA ranged from 4 to 49% of the session, time spent sedentary ranged from 31 to 79%, and screen time accounted for 0 to 41%. MVPA was inversely associated with total sedentary time (*p* < 0.001). Higher screen time was associated with OSHC services being larger in size (*p* = 0.04), offering screen activities on a daily basis (as opposed to less than daily; *p* = 0.001), offering screen activities prior to 5 pm (as opposed to offering screen activity 5 pm or later; *p* = 0.02), and having a larger number of screen devices available (*p* = 0.08).

**Conclusion:**

Physical activity and screen time practices in OSHC services are currently ad hoc and variable. In future, development of guidelines, policy and intervention programs may help improve physical activity and screen time in the OSHC setting.

**Electronic supplementary material:**

The online version of this article (10.1186/s12887-019-1653-x) contains supplementary material, which is available to authorized users.

## Background

Physical activity is fundamentally important to children’s health and wellbeing. Worldwide, a lack of physical activity has been identified as the number one leading cause of premature death and is a significant risk factor for non-communicable disease such as stroke, diabetes, and cancer [1]. Adopting positive health behaviours at a young age has been reported to have a positive impact on growth, development and general health. Habits established in childhood lay the foundation for health into adulthood [2–5]. In addition, higher levels of recreational screen time have been associated with unhealthy eating, increased risk of obesity, increased risk of depressive symptoms and reduced sleep duration and quality in children [6–9].

Despite these well recognised links with health, the majority of children in Australia and other developed countries fail to meet the daily physical activity and screen time guidelines. The most recent global matrix 3.0 demonstrated that in very high-income countries (e.g. US, Canada, Australia, New Zealand, UK, Belgium, Hong Kong, Japan, UAE) only 20–26% of children aged 5–17 years are achieving the recommended amount of moderate-to-vigorous physical activity (MVPA) [10].

The current physical activity guidelines for children aged 5–17 in Australia and internationally recommend that children accumulate at least 60 min of MVPA, and that recreational screen time should be limited to no more than 2 h per day [11–14]. The guidelines are operationalised across the entire day, and do not give recommendations for specific timeslots of the day. In addition to national guidelines, some jurisdictions have physical activity policies stipulating a minimum amount of physical activity (for example, through mandatory minimum weekly amounts of physical education curriculum), and there is evidence such policies positively effect physical activity participation [15]. Another timeslot of the day - the after-school period (approximately 3 pm – 6 pm) - is viewed as being particularly important because it is relatively discretionary, and activities undertaken in this timeslot play a key role in determining whether children meet or fail daily movement guidelines, coining the term the “critical window” [16].

In Australia, approximately 1 in 10 school-aged children attend formal before- and after-school child care services, also known as “out of school hours care” (OSHC) [17]. Children typically attend these services due to their parents’ or guardians’ work or study commitments, and the services set out to provide children with supervised recreational and leisure activities [18]. There are relatively few studies, either within Australia or internationally, that have examined children’s physical activity and sedentary behaviours in the out of school childcare context. In the Australian after-school care setting, Thompson et al. [19] surveyed the nutrition and physical activity practises in 426 OSHC services in the state of Victoria. Active games were reported in 62% of the participating services. However, sedentary behaviours were also common, with 37% of centres using screen-time for a large proportion of the session. Internationally, most work has been conducted in the USA. Beets’ study in South Carolina after-school programs [20] found that children spent the majority of the session sedentary, and only a relatively small amount of the session engaged in MVPA (16–20%). Similarly, Trost et al. [21] reported that children attending after-school programs in the mid-western states of the USA received MVPA for 18% of the session. This contrasts with data presented by Coleman et al. [22], which suggested that children attending after-school programs in Kansas spent 51–69% of the session in MVPA [23].

Given the conflicting evidence, lack of attention given to screen time practices in after-school programs, and lack of recent Australian data, further research to expand and update our understanding of physical activity and screen time in out of school care programs is warranted. This study aimed to address this gap, by examining current physical activity and screen time practises and policies in Australian OSHC services. In addition, it aimed to identify factors that influence physical activity and screen time practises. This will form a crucial first step in identifying priorities for future efforts to promote healthful physical activity and screen time practises in this setting.

## Methods

### Study design

This observational, cross-sectional study was approved by the University of South Australia Human Research Ethics Committee, Flinders University Social and Behavioural Research Ethics Committee, the South Australian Department for Education and Child Development (DECD), and the Catholic Education Office of South Australia. All directors of participating OSHC centres provided written informed consent, and parents of children attending the centres were provided with study information and the opportunity to opt-out.

### Setting

The study involved observation at OSHC services in metropolitan Adelaide, South Australia, with data collection taking place between May and September 2016.

### Participants

A list of all OSHC centres in Adelaide and surrounds was sorted into socio-economic status tertiles based on the schools’ ‘Index of Community Socio-Educational Advantage’ (ICSEA) rating [24]. The ICSEA is a numerical scale (mean of 1000), based on a number of factors including; geographical location, proportion of Indigenous students, parental occupation and level of parental education. A lower ICSEA rating represents a lower level of socio-economic advantage. An equal number of centres from each tertile were randomly selected using a computerised random number generator. The director of each centre was mailed an invitation letter with information outlining the study and followed up with a phone call approximately two weeks later. At each participating OSHC service, participants included the service director, as well as the children attending the OSHC service on the day of data collection.

### Variables/data sources

#### Directors’ survey

Directors completed a 15 min survey via structured interview. The interview items were informed by the ‘Healthy Afterschool Activity and Nutrition Documentation’ (HAAND) instrument [25], and sought information relating to the OSHC service size, policies and practises relating to physical activity and screen time, activities offered and daily activity schedule, availability of equipment for physical activity and screen time, rules regarding physical activity and screen time, and staff training (Additional file 1).

#### Physical activity, sedentary behaviour and screen time

Children’s MVPA, light physical activity, sedentary behaviour and screen time throughout a full after-school session were directly observed using the System for Observing Play and Leisure Activity in Youth (SOPLAY) direct observation tool [26]. Prior to each period of data collection, two research personnel mapped areas accessed by the OSHC students into zones, in collaboration with the OSHC director. Upon commencement of the after-school OSHC session, each zone was visually scanned from left to right with boys and girls recorded separately (one researcher observed girls, and the other observed boys). The intensity of children’s activities was recorded using a 3-way electronic counter as either: sedentary (lying down, sitting or standing, for example, seated video games), walking (e.g. walking to another activity), or vigorous (e.g. active sport such as basketball). In addition, for each visual sweep, the activity type was recorded (e.g. “indoor arts and crafts”, “basketball” etc). All zones were continuously scanned in sequence until there were less than five children remaining at the OSHC service. As recommended by Saint-Maurice et al., walking was re-categorised as light physical activity and vigorous re-categorised as MVPA for analysis [23]. Categorised in this way, there is excellent agreement between MVPA estimated by the SOPLAY relative to accelerometry (mean per cent difference 1.29, SD 9.8) [23].

#### Staff behaviour for supporting physical activity

Staff behaviours enabling or inhibiting children’s physical activity were collected using the System for Observing Staff Promotion of Activity and Nutrition (SOSPAN) direct observation tool [27]. This tool was specifically designed to be used as an adjunct to SOPLAY [27]. After each SOPLAY visual scan, a second scan was performed documenting the accessibility and usability of the area, whether or not there was adult supervision/involvement and/or organised activity and equipment availability. Staff behaviour in relation to physical activity was categorised as either being engaged, off task, performing another duty, instructing, promoting, discouraging, withholding or punishing [27]. The SOSPAN has demonstrated validity and good reliability, with inter-observer agreement ranging from 75 to 100% [27].

### Bias

A number of steps were taken to minimise bias. Firstly, a computerised random number generator was used to identify OSHC services to be invited to the study, stratified by SES, to recruit a representative sample. Directors were assured that their service would not be identified in reporting of results and instructed that there were no “right” or “wrong” answers, to minimise the potential for social desirability bias. All research personnel received comprehensive training in administration of the SOPLAY and SOSPAN instruments, including completing online SOPLAY training modules [28], and participating in two practice visits prior to the main data collection for the study. Use of opt-out consent for child participants meant that very high (100%) participation was achieved, increasing confidence that the observed behaviours reflect the OSHC student population.

### Sample size

Due to the descriptive aims of the study, formal power calculations were not undertaken. At the study’s inception, the research team deliberated the target sample size, and agreed to aim for a target sample of ≥20 OSHC services, which was deemed sufficient to capture variability among OSHC services, and also feasible given resource constraints.

### Procedure

A date and time were arranged to visit each participating OSHC centre during the after-school care period. Fridays were avoided because formative work indicated that less children were likely to be attending, and programming on that day may be different to the rest of the week. Visits were rescheduled if there was a moderate or high chance of rain forecast during the after-school period. All participating OSHC directors were emailed parent information letters and opt-out consent forms were provided to all parents/caregivers of the children due to attend OSHC on the date of the scheduled visit.

On the day of data collection, two research personnel attended the participating centre for an entire afternoon. During the visit, they met with the OSHC director, who provided written informed consent, and participated in the survey interview. In addition, they prepared for the observational component of data collection, and observed the children and staff for the full after-school session (approximately 3:00 pm to 6:00 pm).

### Statistical analysis

Data were compiled in Microsoft Excel and analysed using Excel and SPSS (v.24, IBM). Closed-ended survey items (for example, number of students enrolled, and existence of a physical activity policy) were analysed using descriptive statistics including means, standard deviations, frequencies, percentages and ranges. Open-ended survey items were categorised into common themes. In some cases where it was possible and meaningful, responses were converted to frequency. For example, many directors reported implementing rules restricting recreational screen time. Such data were re-coded to determine whether each OSHC service offered screens daily, content-based limitations, context-based limitations (e.g. bad weather only), and time restrictions. Children’s observed physical activity and sedentary behaviour data recorded using SOPLAY were used to calculate the percentage of each after-school session spent in each movement behaviour category (MVPA, light physical activity, and sedentary time) for each OSHC service. This approach accounted for the fact that the number of children in attendance gradually reduced across the duration of the care session, due to children being collected by their parents/guardians. Sedentary behaviour was further broken down into non-screen sedentary time and screen-based sedentary time, with screen-based time categorised by type of device (seated video games, iPads/tablets/mobile phones/handheld devices, television and DVDs). Similar to the SOPLAY data, staff behaviour data collected using the SOSPAN (enabling/disabling physical activity) were collapsed to produce a percentage of each after-school session spent in PA enabling behaviours (instructing, engaging or promoting physical activity), passive behaviours (staff off task, or on other duties) and PA disabling behaviours (withholding of physical activity or using physical activity as a punishment). Finally, hypothesis generating analyses examining the relationships between MVPA and screen time and possible predictors were undertaken using stepwise backward linear regression. For these analyses, the percent (%) of the session spent in MVPA or screen time was used as the dependent variable respectively. Both of these variables were right-skewed, therefore they were log-transformed to normalise their distribution. The predictor variables used in the models were: size of OSHC service (i.e. number of students in attendance), staff behaviours enabling and disabling physical activity, whether staff facilitated physically activity games (Y/N), duration of outdoor play offered, number of active play zones available, total number of screen devices available, daily screen availability (Y/N), and availability of screens before 5 pm (Y/N). Analyses were conducted in SPSS. Due to the exploratory nature of these analysis and limited sample size, alpha was set at *p* < 0.10.

## Results

### Participants

A total of 53 directors were mailed introductory letters and phoned to discuss possible participation, of whom 23 (44%) agreed to participate. A total of *n* = 1068 children attended OSHC on the days of data collection (ranging from 8 to 114 at individual centres (mean: 46, SD: ±25). Of the 23 participating centres, 18 were based in government schools, and the remaining five were located in Catholic schools. The mean (SD) ICSEA (Index of Community Socio-Educational Advantage) score for participating services was 1045 (SD 57), which closely matched the ICSEA values for all OSHC services in Adelaide and surrounds (1046 (64)), as well as the services that were invited to participate in the study but declined (1046 (65)).

### Descriptive data

#### Observed physical activity and sedentary behaviour

**Fig. 1:**
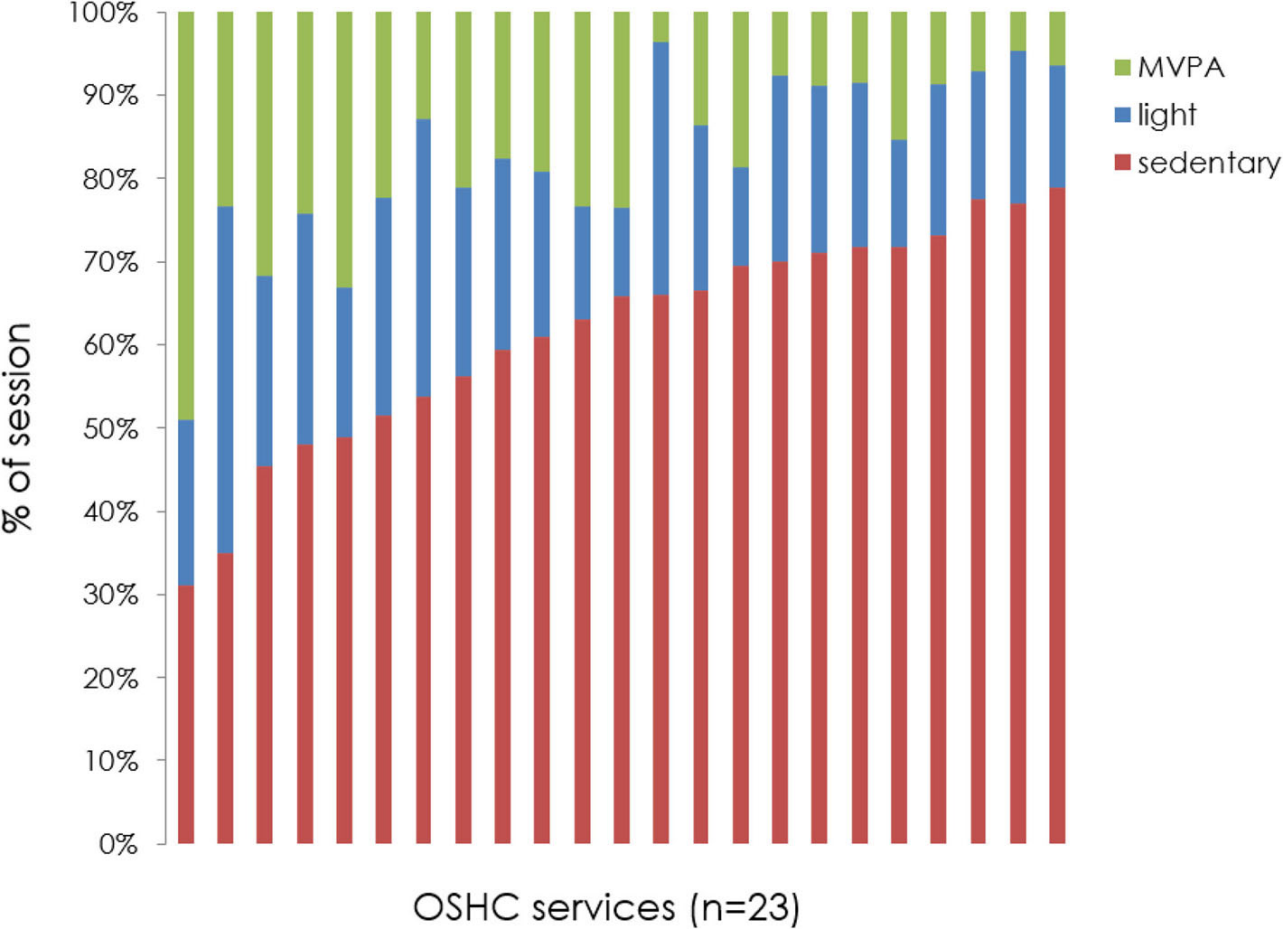
% of session spent in physical activity behaivours at OSHC services

On average across all OSHC services, children spent 61% (SD 15%) of the OSHC session in sedentary behaviour, 21% in light physical activity (SD 9%) and 18% in MVPA (SD 12%). However, the amount of time spent in activity bands was highly variable among OSHC services (Fig. 1). For example, sedentary behaviour accounted for between 31 and 79% of the session at different OSHC services, whilst MVPA accounted for between 4 and 49% of the session.

**Fig. 2:**
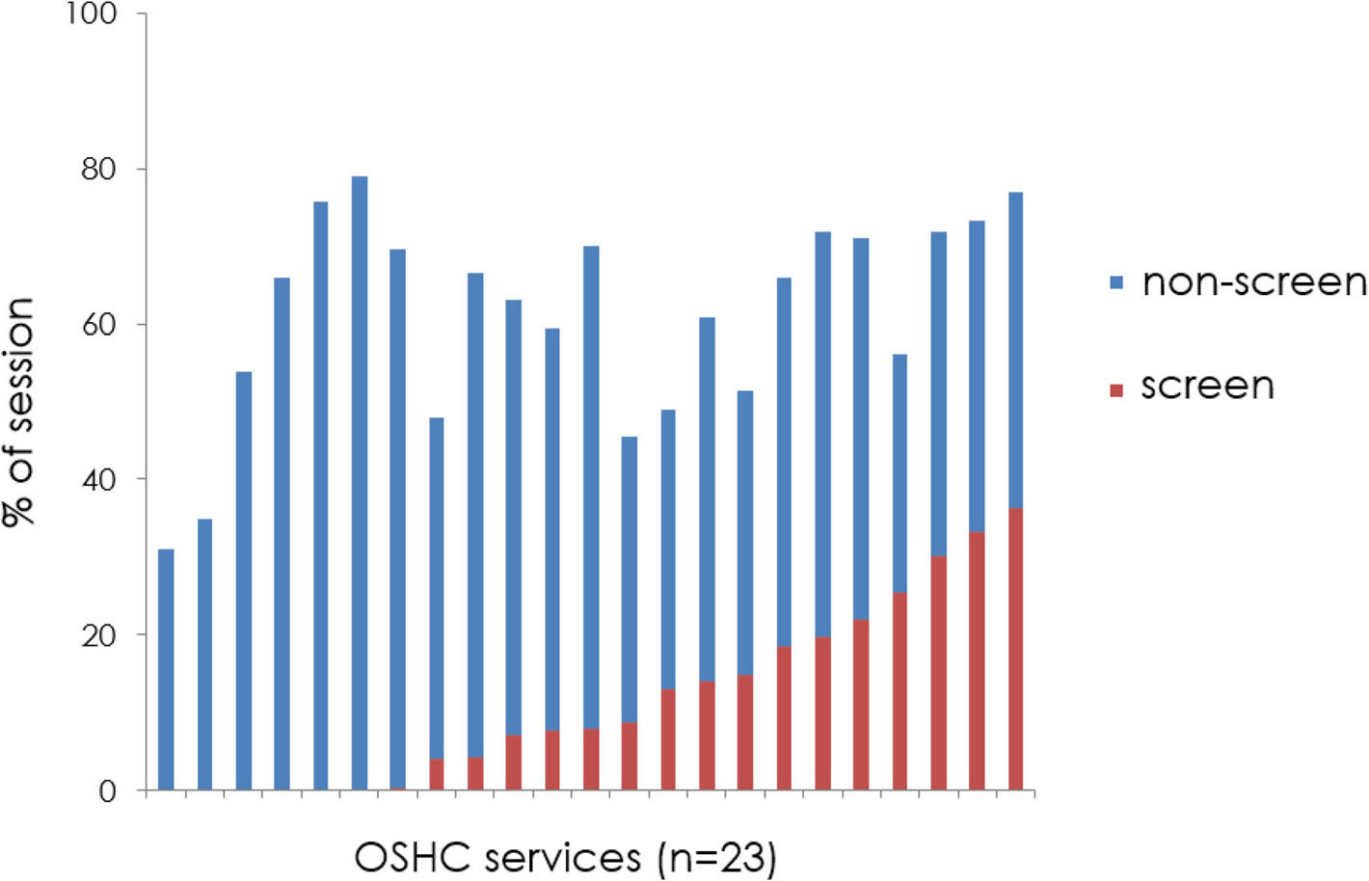
% of session in sedentary behaviour non-screen vs screen time

The three most common MVPA activities were 1) unstructured play, 2) playground equipment play, and 3) soccer. Screen time accounted for an average of 17% of total session time. However, this was highly variable amongst the different services (Fig. 2), ranging from none in some services (*n* = 6) to 41% in one service. Four of the centres had children participating in screen time for over 30% of the session. The most common forms of screen time were passive video games, mobile devices (i.e. iPad/tablet/mobile phone), and television viewing.

#### Staff behaviour

Staff behaviours related to enabling/disabling physical activity were observed throughout the OSHC sessions. Most commonly, staff members were involved in “other duties” whilst children played (63% of total behaviours). Around one third of the time (36%), staff members displayed positive behaviour, either through engaging with students in physical activity (16%), promoting physical activity (12%) or instructing students regarding physical activity (11%). Negative staff behaviours related to physical activity were uncommon (e.g. using physical activity as a punishment was never observed, and withholding physical activity (i.e. being made to sit out of a play activity as a behaviour management strategy) was observed < 0.001% of the time).

### Directors’ survey

#### Policy and staff training

Directors were asked about policies and staff training relating to physical activity and screen time in their centre. Around one third (39%) of centres replied affirmatively when asked whether they had a physical activity policy, however they typically paused before answering this question (suggesting they were unsure), and some clarified that they were answering yes on the basis that there was a policy for children’s physical and emotional health more broadly, which they perceived to encompass physical activity, as opposed to having a specific physical activity policy. Two thirds (70%) stated that their staff were trained in relation to physical activity, primarily through training that was incurred outside their roles as OSHC staff (e.g. it was common for OSHC staff to be university students studying teaching or health-related degrees).

#### Activities offered and session structure

**Fig. 3:**
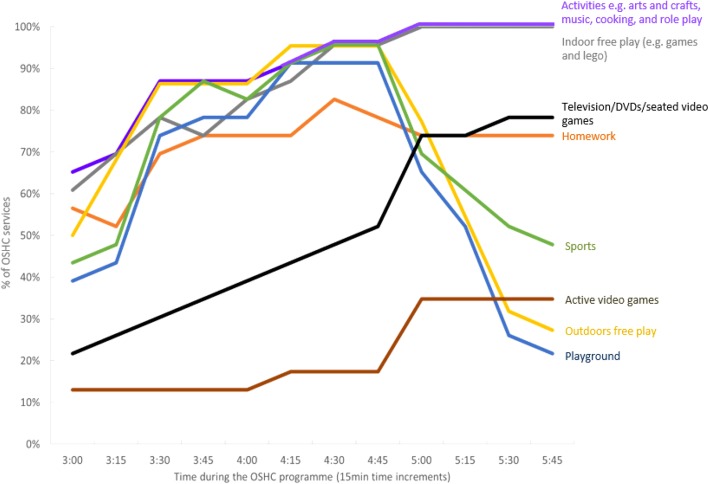
The percentage of OSHC services offering various activities at different timeslots (15 min increments) within an afternoon care session

A wide variety of activities were offered by the OSHC centres, such as drawing and craft, inside play (e.g. using toys such as Lego), outside play, use of sports equipment, homework time and recreational screen time. However, the way in which the activities were offered varied among services. For example, it was common for indoor sedentary activities such as drawing and crafts, inside play (e.g. with Lego) and homework, to be offered throughout the whole session. Activities conducive of MVPA (e.g. sports and playground play) were typically offered for a limited period early in the session (e.g. from 3:30 to 4:45 pm), while screen-based activities were relatively uncommon in the first half of the session but increasingly common from 4:45 pm onwards (Fig. 3). With regards to timetabling for physical activity, one third (30%) of services reported that they strongly encouraged children to engage in MVPA (for example, by requiring children to play outside for part of the session), whilst the other two thirds reported that they offered active play as one option alongside other activity options (e.g. inside play and craft).

#### Screen time equipment and practices

All OSHC services, with a single exception, reported offering recreational screen time, typically using devices supplied by the OSHC service (e.g. television, iPads, videogame consoles and computers). Some services reported recreational screen use on a daily basis (65%), whereas others reported it weekly (e.g. on a Friday), or only occasionally (e.g. rainy day, or last week of term). The majority of directors reported their service enforced rules to restrict screen time, typically related to daily scheduling (e.g. screens could only be used after 5 pm; reported by 39% of services), time limits (e.g. each student could play a video game for 15 min; reported by 13%), or content viewed (see Additional file 2).

#### Factors associated with favourable/unfavourable physical activity and screen time practises

Overall, there was a strong inverse relationship between MVPA and total sedentary time (r = − 0.835, *p* < 0.001), while there was only a weak, non-significant correlation between MVPA and screen time (r = − 0.25, *p* = 0.26).

Exploratory regression analyses were undertaken to identify characteristics associated with physical activity and screen time behaviour. The physical activity model showed a poor fit (adjusted R-square for the full model = − 0.37). None of the hypothesised predictors were significantly associated with MVPA behaviour in the full model (shown in Table 1), and all predictors were excluded from the final (best-fit) model.

**Table 1:** Regression analysis examining the relationship between % of session in MVPA and potential predictors (full model shown)

	Standardised beta	t	*p*
OSHC service size	−0.12	− 0.39	0.70
Staff behaviour enabling PA	−0.15	−0.47	0.65
Staff behaviour disabling PA	0.15	0.51	0.62
number of active play zones available	−0.14	−0.49	0.63
whether staff facilitated physically activity games (Y/N)	0.00	0.01	0.99
duration of outdoor play offered	−0.04	−0.12	0.90
Total number of screen devices	−0.27	−0.74	0.48
daily screen availability (Y/N)	−0.61	−0.96	0.35
availability of screens before 5 pm (Y/N)	0.10	0.26	0.80

In contrast, the regression model was able to predict around 60% of variation in screen time behaviours (adjusted R-square for full model and best-fit models = 0.58 and 0.66 respectively, see Table 2). Higher screen time was associated with OSHC services being larger in size, offering screen activities on a daily basis (as opposed to less than daily), offering screen activities prior to 5 pm (as opposed to offering screen activity 5 pm or later), and having a larger number of screen devices available.

**Table 2:** Regression analysis examining the relationship between % of session in screen time and potential predictors

	Standardised beta	t	*p*
FULL MODEL			
OSHC service size	0.36	1.68	0.14
Staff behaviour enabling PA	0.46	1.64	0.15
Staff behaviour disabling PA	−0.06	− 0.32	0.76
number of active play zones available	−0.10	−0.45	0.67
whether staff facilitated physically activity games (Y/N)	−0.43	−1.37	0.22
duration of outdoor play offered	0.28	1.06	0.33
Total number of screen devices	0.88	2.77	0.03
daily screen availability (Y/N)	0.67	2.52	0.05
availability of screens before 5 pm (Y/N)	−0.43	−1.79	0.12
BEST FIT MODEL			
OSHC service size	0.38	2.40	0.04
daily screen availability (Y/N)	0.84	4.44	0.001
Total number of screen devices	0.48	2.86	0.02
availability of screens before 5 pm (Y/N)	−0.39	−1.91	0.08

## Discussion

This study aimed to describe current practices and policies regarding physical activity and screen time in OSHC services. Results revealed a lack of formal policies regarding physical activity and screen time. On average, children spent around one fifth of the OSHC sessions engaging in MVPA, whilst two thirds were spent sedentary. Recreational screen activities accounted for around 40% of sedentary activity (which overall equated to 17% of the entire OSHC session). Importantly, results revealed that the amount of the session children spent in MVPA or sedentary and screen activities varied widely among OSHC services – in some services children engaged in virtually no MVPA, and in some services, children spent over 40% of the session on screens. Exploratory analyses did not identify any significant predictors of MVPA behaviours, however they did suggest that screen behaviour was higher in larger OSHC services, services that had more screen devices, and services that allowed daily screen activity (and particularly services that allowed screen activities prior to 5 pm).

The findings regarding activity patterns in the OSHC setting in this study are reasonably consistent with previous research. For example, both Huberty et al. [29] and Thompson et al. [19] reported that around two thirds of children’s time was spent sedentary, which is similar to the rate observed in our study. In addition, the rate of MVPA was similar to, or slightly lower than that reported in previous studies. For example, we found MVPA for 18% of the session, which is similar to rates reported by Beets et al. [20] and Trost et al. [21], but somewhat lower than that reported by Coleman et al. [22] and Thompson. [19] It is possible that the lower rate observed in our study was due to measurement differences – given that Thompson et al. used self-reported data, and Coleman used the original SOPLAY instrument, whereas we used SOPLAY with the updated scoring method recommended by Saint Maurice et al. [23].

We attempted to identify which factors predicted favourable/unfavourable MVPA and screen time practices. Our statistical analyses failed to identify any significant predictors for MVPA, while lower screen time was associated with the OSHC services being smaller in size (i.e. fewer enrolled students), services offering fewer screen devices, and restricting of recreation screen use through timetabling. Previous research has suggested that higher MVPA in OSHC programs is associated with availability of physical activity equipment, as well as the staff not being involved in other behaviours or off task [29]. It has also been suggested that providing children with free-play opportunities can favourably impact MVPA behaviour in out of school care [29, 30]. Whilst this study did not confirm these findings, some of our observations were broadly consistent with this. For example, free-play was the most popular form of MVPA we observed. Unexpectedly, the OSHC service which we found to have the *lowest* level of MVPA, was remarkable in that it provided adult-led games and physical activities (e.g. tunnel ball and yoga) for approximately half the afternoon care session. Thus in this particular centre, it seems that having adults-led activities, may have had the opposite effect to that intended. This is consistent with Coleman and colleagues’ study [22] which suggested that children were significantly more active during free play sessions compared with organised adult-led sessions. This may suggest that there is a need for higher quality professional development for staff to help them effectively facilitate physical activity.

### Strengths and limitations

This study was novel both internationally and in Australia specifically, given the lack of knowledge about screen behaviours in after-school care internationally, and lack of up-to-date evidence for either physical activity or screen behaviours in Australian OSHC. The current study used a standardised direct observation methodology to measure physical activity and screen time, which is considered gold standard, and allows contextual information to be captured which is not possible with other measurement methods such as accelerometry. A large number of children participated (*n* = 1068), and the participation rate achieved for children was excellent (100%), reducing the possibility of selection bias. The OSHC service participation rate of 23/53 (43%) is comparable to previous school-based research [6]. A limitation of the study was that it was only conducted in a single city, thus it is unclear whether results are generalisable to rural areas and other cities. In each OSHC service, data were collected for a single after-school session. Whilst services were encouraged to continue with their normal programming, it is possible that they may have modified their activities due to awareness that they were being observed. In the director’s survey, directors sometimes reported on their staff’s participation in physical activity-related training incurred in roles independent of their employment as OSHC staff, and it is not possible to confirm whether this training indeed took place. OSHC visits only occurred on days when rain was not forecast, thus findings are not generalisable to rainy days. For the exploratory analyses examining possible predictors of MVPA and screen time, the OSHC service was the unit of analysis (*n* = 23), and thus these analyses had limited statistical power. While we increased the alpha to 0.10 in an attempt to compensate for this, there is still a risk of type 2 errors (i.e. failure to detect relationships that truly exist).

#### Future directions

To date, the physical activity and screen-time behaviours in after-school care settings have received relatively little attention to other day segments and contexts. However, given the large number of children attending these programs, and the discretionary nature of activities undertaken during this time window, the potential for capitalising on OHSC to positively impact children’s daily activity patterns appears vast. This study highlighted that physical activity and screen time are currently largely influenced by “in-house” factors, with a lack of specific guidance from government policy. During the structured interviews with the service directors, many expressed that they felt uncertain what they should be doing with regards to physical activity and screen-based activities and reported they would welcome future directives. In some jurisdictions, physical activity guidelines specific to after-school care settings have been published. For example, the Californian Department of Education [31], which was adopted by the National Afterschool Association in the US [32] recommends that children should get at least 30 min of MVPA during an afternoon care session. Similarly, the Ontario Ministry of Education [33] adopted a similar policy which recommends “…a minimum of 30 minutes active play in daily timetabling….and to avoid leisure screen time” [33]. Recent work in South Carolina suggests that provision of OSHC guidelines for physical activity, combined with an implementation intervention, achieved some success in increasing children’s physical activity levels [34].

Further work quantifying the amount of MVPA (e.g. using accelerometry) and screen time obtained in OSHC in terms of daily minutes would be beneficial. This would allow direct comparison to children’s daily MVPA and screen time guidelines, would be beneficial to confirm whether future programs aimed at increasing MVPA/reducing screen time in the OSHC setting are required. Certainly, in the past 10 to 15 years in Australia, some efforts have been made to increase MVPA in OSHC, in particular through two nationally-funded programs which involved third party providers visiting OSHC services to run regular sports/physical activity [35, 36]. Unfortunately, both of these programs have been subsequently abandoned, due to the high cost of implementation, combined with a lack of evidence of effectiveness [35, 36]. It seems possible that low cost strategies may be more sustainable and still positively impact activity patterns. For example, the strong inverse correlation between MVPA and sedentary behaviour identified in our study suggests that limiting availability of sedentary activities might positively impact MVPA. Further avenues for improving activity patterns suggested by our results would be maximising opportunities for free play, and restricting access to screen-based activities. Future work using an implementation science approach, focussing on understanding intervention effectiveness and feasibility, and prioritising potential for scale-up, is needed [37].

## Conclusion

In conclusion, most children, both in Australia and in many countries globally, do not get enough physical activity and get too much screen time, and OSHC setting offers an opportunity to positively impact the daily activity patterns of children. This study highlighted that current practises regarding physical activity and screen time in the OSHC setting are highly variable across services, and that there is lack oversight/guidance from overarching guidelines and policy. Research in the out of school care setting is scant, and future work examining practice and policy in other jurisdictions will be important to determine the scale of the issue. Development of guidelines and intervention programs to encourage healthful physical activity and screen behaviours in the out of school care setting appears warranted.

## Additional files

Additional file 1: The OSHC directors' survey. (DOCX 49 kb)

Additional file 2: Examples of the different rules that schools enforced, some OSHC centres may enforce more than one rule at one time. (DOCX 16 kb)

## Data Availability

The datasets used and/or analysed during the current study are available from the corresponding author on request.

## Abbreviations

DECD: Department for Education and Child Development
HAAND: Healthy Afterschool Activity and Nutrition Documentation
ICSEA: Index of Community Socio-Educational Advantage
MVPA: Moderate-to-Vigorous Physical Activity
OSHC: Out of School Hours Care
SES: Socio-Economic Status
SOPLAY: System for Observing Play and Leisure Activity in Youth
SOSPAN: System for Observing Staff Promotion of Activity and Nutrition
SPSS: Statistical Package for the Social Sciences
