# Supplementary material for: Physical activity and screen time in out of school hours care: an observational study
Source: BMC Pediatr. 2019 Aug 14;19:283. doi: 10.1186/s12887-019-1653-x (PMC6693127; doi:10.1186/s12887-019-1653-x)
Supplement: Supplementary file 1 — The OSHC directors' survey. (DOCX 49 kb) [file 12887_2019_1653_MOESM1_ESM.docx]

SUPPLEMENTARY FORM – Directors Survey


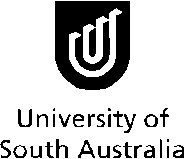

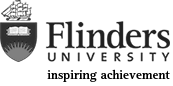


School _____________________ Date ________ Research assistant name _______________________

**Physical Activity and Nutrition in Out of School Hours Care**

**Director Survey**

**1. Does your Service have a written PHYSICAL ACTIVITY policy specific for OSHC?**

○ No

○ No, but we are in the process of developing a written policy

○ Yes

○ Other (please specify)

_______________________________________________________________________________________

**2. Does your Service have a written NUTRITION policy specific for OSHC?**

○ No

○ No, but we are in the process of developing a written policy

○ Yes

○ Other (please specify)

_______________________________________________________________________________________

**3. Does your Service formally schedule a certain amount of time in the after school care program for PHYSICAL ACTIVITY?** Please provide details…

______________________________________________________________________________________________________________________________________________________________________________

**4. Do staff at your Service complete any TRAINING in physical activity or healthy eating?**

○ Staff are not trained in physical activity or healthy eating practices

○ Staff are trained in physical activity

○ Staff are trained in healthy eating

○ Staff are trained in BOTH physical activity and healthy eating

Please provide the relevant details of any staff training here (including the provider/s and approximate time commitment per year)…

______________________________________________________________________________________________________________________________________________________________________________

**5. Please tick whether the following ACTIVITIES are offered at after school care at your Service (first column).**

**If the activity is offered, please indicate the approximate time(s) for a ‘typical day’** (if you are unsure of the times, please indicate ‘don’t know’)

| Activity | **Offered** | 3:00 – 3:15pm | 3:15 – 3:30pm | 3:30 – 3:45pm | 3:45 – 4:00pm | 4:00 – 4:15pm | 4:15 – 4:30pm | 4:30 – 4:45pm | 4:45 – 5:00pm | 5:00 – 5:15pm | 5:15 – 5:30pm | 5:30 – 5:45pm | 5:45 – 6:00pm | Don’t know |
| --- | --- | --- | --- | --- | --- | --- | --- | --- | --- | --- | --- | --- | --- | --- |
| Arts + crafts | **○** | ○ | ○ | ○ | ○ | ○ | ○ | ○ | ○ | ○ | ○ | ○ | ○ | ○ |
| Homework / reading (with assistance) | **○** | ○ | ○ | ○ | ○ | ○ | ○ | ○ | ○ | ○ | ○ | ○ | ○ | ○ |
| Homework / reading  (no assistance) | **○** | ○ | ○ | ○ | ○ | ○ | ○ | ○ | ○ | ○ | ○ | ○ | ○ | ○ |
| TV / DVD | **○** | ○ | ○ | ○ | ○ | ○ | ○ | ○ | ○ | ○ | ○ | ○ | ○ | ○ |
| iPads / tablets | **○** | ○ | ○ | ○ | ○ | ○ | ○ | ○ | ○ | ○ | ○ | ○ | ○ | ○ |
| Computer | **○** | ○ | ○ | ○ | ○ | ○ | ○ | ○ | ○ | ○ | ○ | ○ | ○ | ○ |
| Video games (sitting down) | **○** | ○ | ○ | ○ | ○ | ○ | ○ | ○ | ○ | ○ | ○ | ○ | ○ | ○ |
| Video games (standing up e.g. Wii) | **○** | ○ | ○ | ○ | ○ | ○ | ○ | ○ | ○ | ○ | ○ | ○ | ○ | ○ |
| Listening to music | **○** | ○ | ○ | ○ | ○ | ○ | ○ | ○ | ○ | ○ | ○ | ○ | ○ | ○ |
| Free play (inside) | **○** | ○ | ○ | ○ | ○ | ○ | ○ | ○ | ○ | ○ | ○ | ○ | ○ | ○ |
| Activity | **Offered** | 3:00 – 3:15pm | 3:15 – 3:30pm | 3:30 – 3:45pm | 3:45 – 4:00pm | 4:00 – 4:15pm | 4:15 – 4:30pm | 4:30 – 4:45pm | 4:45 – 5:00pm | 5:00 – 5:15pm | 5:15 – 5:30pm | 5:30 – 5:45pm | 5:45 – 6:00pm | Don’t know |
| Free play (outside) | **○** | ○ | ○ | ○ | ○ | ○ | ○ | ○ | ○ | ○ | ○ | ○ | ○ | ○ |
| Cooking / food preparation | **○** | ○ | ○ | ○ | ○ | ○ | ○ | ○ | ○ | ○ | ○ | ○ | ○ | ○ |
| Board games / Lego | **○** | ○ | ○ | ○ | ○ | ○ | ○ | ○ | ○ | ○ | ○ | ○ | ○ | ○ |
| Playground (inside) | **○** | ○ | ○ | ○ | ○ | ○ | ○ | ○ | ○ | ○ | ○ | ○ | ○ | ○ |
| Playground (outside) | **○** | ○ | ○ | ○ | ○ | ○ | ○ | ○ | ○ | ○ | ○ | ○ | ○ | ○ |
| Sports equipment play (e.g. balls/hoops) | **○** | ○ | ○ | ○ | ○ | ○ | ○ | ○ | ○ | ○ | ○ | ○ | ○ | ○ |
| Role play / dress ups | **○** | ○ | ○ | ○ | ○ | ○ | ○ | ○ | ○ | ○ | ○ | ○ | ○ | ○ |
| Snack time | **○** | ○ | ○ | ○ | ○ | ○ | ○ | ○ | ○ | ○ | ○ | ○ | ○ | ○ |
| Other (please specify)  ______________________ | **○** | ○ | ○ | ○ | ○ | ○ | ○ | ○ | ○ | ○ | ○ | ○ | ○ | ○ |
| Other (please specify)  ______________________ | **○** | ○ | ○ | ○ | ○ | ○ | ○ | ○ | ○ | ○ | ○ | ○ | ○ | ○ |
| Other (please specify)  ______________________ | **○** | ○ | ○ | ○ | ○ | ○ | ○ | ○ | ○ | ○ | ○ | ○ | ○ | ○ |

**6. Please indicate the approximate number of the following DEVICES available to children in after school care at your Service**

TVs ___________________

DVDs ___________________

iPads / tablets ___________________

Computers ___________________

Video game consoles ___________________

**7. Please outline whether there are any rules or limitations regarding the use of the following DEVICES for children in after school care at your Service**

TVs ____________________________________________________

DVDs ____________________________________________________

iPads / tablets ____________________________________________________

Computers ____________________________________________________

Video game consoles ____________________________________________________

**8. Are children allowed to bring personal electronic DEVICES for use at after school care (e.g. iPods, iPads, gaming devices)?**

○ Yes

○ No

If yes, please specify

______________________________________________________________________________________________________________________________________________________________________________

**9. Please indicate the types + frequency of FOODS that are offered at after school care at your Service**

|  | **Never** | **Occasionally** | **Most days** | **All days** |
| --- | --- | --- | --- | --- |
| White bread / toast / sandwiches | ○ | ○ | ○ | ○ |
| Wholegrain or whole meal bread / toast / sandwiches | ○ | ○ | ○ | ○ |
| Unsalted plain snacks (e.g. plain popcorn) | ○ | ○ | ○ | ○ |
| Salty unflavoured snacks (e.g. pretzels, plain rice crackers) | ○ | ○ | ○ | ○ |
| Salty flavoured snacks (e.g. flavoured chips, Doritos) | ○ | ○ | ○ | ○ |
| Fresh fruit | ○ | ○ | ○ | ○ |
| Dried fruit | ○ | ○ | ○ | ○ |
| Pre-packaged fruit (e.g. tinned fruit) | ○ | ○ | ○ | ○ |
| Fruit roll ups or leathers | ○ | ○ | ○ | ○ |
| Fresh vegetables / salad | ○ | ○ | ○ | ○ |
| Plain yoghurt | ○ | ○ | ○ | ○ |
| Flavoured yoghurt | ○ | ○ | ○ | ○ |
| Cheese (low fat) | ○ | ○ | ○ | ○ |
| Cheese (full fat) | ○ | ○ | ○ | ○ |
| Sweet biscuits / cookies | ○ | ○ | ○ | ○ |
| Cake / bun | ○ | ○ | ○ | ○ |
| Meat | ○ | ○ | ○ | ○ |
| Lollies (chocolate / sweets) | ○ | ○ | ○ | ○ |
| Cereal (sugar sweetened, e.g. Coco Pops) | ○ | ○ | ○ | ○ |
| Cereal (non-sweetened) (e.g. oats, Weet Bix) | ○ | ○ | ○ | ○ |
| Other(s) _______________________________ | ○ | ○ | ○ | ○ |
| Other(s) _______________________________ | ○ | ○ | ○ | ○ |

**10. Please indicate the types + frequency of DRINKS that are offered at after school care at your Service**

|  | **Never** | **Occasionally** | **Most days** | **All days** |
| --- | --- | --- | --- | --- |
| Low-fat plain milk | ○ | ○ | ○ | ○ |
| Full-fat plain milk | ○ | ○ | ○ | ○ |
| Flavoured milk (e.g. chocolate milk, Milo) | ○ | ○ | ○ | ○ |
| 100% fruit juice | ○ | ○ | ○ | ○ |
| Fruit drink (sweetened) | ○ | ○ | ○ | ○ |
| Cordial | ○ | ○ | ○ | ○ |
| Water | ○ | ○ | ○ | ○ |
| Soft drink | ○ | ○ | ○ | ○ |
| Sports drink | ○ | ○ | ○ | ○ |
| Other(s) _______________________________ | ○ | ○ | ○ | ○ |
| Other(s) _______________________________ | ○ | ○ | ○ | ○ |

**11. Are children allowed to bring / consume FOOD from home at after school care at your Service?**

○ Yes

○ No

○ Other (please specify)

______________________________________________________________________________________________________________________________________________________________________________

**12. Are children allowed to bring / consume DRINK from home at after school care at your Service?**

○ Yes

○ No

○ Other (please specify)

______________________________________________________________________________________________________________________________________________________________________________

**Thank you for taking the time to complete this survey**

Office use only

Location: ○ Private OSHC Service

○ Public OSHC Service

○ Located on-site at a primary school

○ Located off-site

OSHC Service name: ___________________________

Date of visit: ____/____/______

No. of attending children: ___________

○ boys

○ girls

No. of staff: ___________

Program start: ____:____pm

Program end: ____:____pm

Research staff attending: __________________________________________________

__________________________________________________

Today’s weather: Approximate temperature at time of visit _________ ^○^C

○ Sunny

○ Part sun / part cloud

○ Cloudy

○ Rainy
