# Supplementary material for: Physical activity and screen time in out of school hours care: an observational study
Source: BMC Pediatr. 2019 Aug 14;19:283. doi: 10.1186/s12887-019-1653-x (PMC6693127; doi:10.1186/s12887-019-1653-x)
Supplement: Supplementary file 2 — Examples of the different rules that schools enforced, some OSHC centres may enforce more than one rule at one time. (DOCX 16 kb) [file 12887_2019_1653_MOESM2_ESM.docx]

Additional file 2 - **Examples of the different rules that schools enforced, some OSHC centres may enforce more than one rule at one time**

| **Rules for TV**  ***n*=19** | ***n*=** | **Rules for DVD *n*=18** | ***n*=** | **Rules for iPad *n*=13** | ***n*=** | **Rules for Computers *n*=17** | ***n*=** | **Rules for Video Games *n*=13** | ***n*=** |
| --- | --- | --- | --- | --- | --- | --- | --- | --- | --- |
| *Time dependent:* | | | | | | | | | |
| From 1700/1730 onwards, before 1600 | 5 | From 1700/1730 onwards, before 1600 | 5 | From 1600 onwards | 1 | From 1600/1700 onwards | 5 | From 1600/1700 onwards | 3 |
| 15-minute turns: max 2 turns in a row | 1 | Only 1 movie per session | 1 | Rotation: 5-30mins at one time. | 9 | A max. set of time (e.g. 1hr or 20mins) | 2 | A max. of 2 turns in a row | 1 |
|  |  |  |  |  |  | Rotation: 10-30 mins at one time | 7 | Rotation: 5 -30 mins at one time | 9 |
|  |  |  |  |  |  | A max. of 2 turns in a row | 1 |  |  |
| *Day dependent:* | | | | | | | | | |
| Some Fridays | 1 | Only on Fridays | 1 | Only 1 day a week | 1 | 1 night per fortnight | 1 | 1 night per week | 1 |
|  |  | Only on special occasions | 1 |  |  | 4 days per week | 1 |  |  |
|  |  |  |  |  |  | Alternate days | 1 |  |  |
|  |  |  |  |  |  | Roster | 1 |  |  |
| *Weather dependent:* | | | | | | | | | |
| Wet weather only | 5 | Wet weather only | 5 |  |  | Dependent on weather | 1 | Dependent on weather | 1 |
| *Staff Discretion:* | | | | | | | | | |
| Staff monitor it | 1 | Staff monitor it | 1 | Staff iPad - only allowed to use if desperate | 1 | Used as an incentive for homework | 1 | As staff allow | 1 |
| As staff allow | 2 | As staff allow | 1 | As staff allow | 1 | As staff allow | 1 |  |  |
| *Rules regarding content:* | | | | | | | | | |
| G/PG only/Kids channels | 7 | G & PG (with parental permission) | 8 | Educational use only - internet is monitored | 1 | Educational use only | 2 | G/PG | 1 |
| Movies only | 1 |  |  |  |  | Non-violent only | 1 |  |  |
| *Miscellaneous*: | | | | | | | | | |
|  |  |  |  | Year 5+ may bring personal devices for homework | 1 | Only older children | 1 |  |  |
